# Optic Nerve Sheath Meningiomas: Solving Diagnostic Challenges with ^68^Ga-DOTATOC PET/CT

**DOI:** 10.3390/diagnostics13132307

**Published:** 2023-07-07

**Authors:** Tatiana Horowitz, Betty Salgues, Laetitia Padovani, Kaissar Farah, Henry Dufour, Olivier Chinot, Eric Guedj, Thomas Graillon

**Affiliations:** 1 Nuclear Medicine Department, APHM, CNRS, Centrale Marseille, Institut Fresnel, Timone Hospital, CERIMED, Aix Marseille University, 13005 Marseille, France; betty.salgues@aphp.fr (B.S.); eric.guedj@ap-hm.fr (E.G.); 2 Radiotherapy Department, APHM, Timone Hospital, 13005 Marseille, France; 3 Neurosurgery Department, INSERM, MMG, APHM, Timone Hospital, Aix-Marseille University, 13005 Marseille, France; kaissar.farah@ap-hm.fr (K.F.); henry.dufour@ap-hm.fr (H.D.); thomas.graillon@ap-hm.fr (T.G.); 4 Neuro-Oncology Department, APHM, Timone Hospital, 13005 Marseille, France; olivier.chinot@ap-hm.fr

**Keywords:** meningiomas, meningioma, ONSM, optic nerve sheath meningioma, PET, DOTATOC, orbital tumour, SSTR

## Abstract

^68^Ga-DOTATOC PET could be a noninvasive, highly sensitive, and specific technique for the challenging diagnosis of optic nerve sheath meningioma (ONSM). Our objective was to report the use and results of ^68^Ga-DOTATOC PET in suspected ONSM. Twelve subjects who underwent ^68^Ga-DOTATOC PET for suspected ONSM in our department were retrospectively included. Standardised clinical and radiological data were collected. The PET examination results were classified as positive or negative, and lesion standardised uptake values (SUV_max_) were recorded. ^68^Ga-DOTATOC PET confirmed positive uptake in six cases (SUV_max_ > 5), leading to ONSM diagnoses followed by radiation therapy in patients with vision loss. Six ^68^Ga-DOTATOC PET scans were considered negative (SUV_max_ < 5); these comprised one case of neurosarcoidosis, one cavernous malformation, and four uncertain diagnoses, leading to further investigation. ^68^Ga-DOTATOC PET was helpful in tumour volume delineation before radiation therapy, leading to a decrease in dose exposure. Noninvasive ^68^Ga-DOTATOC PET should be performed before treating nonhistologically proven meningiomas with radiotherapy or stereotactic radiosurgery, particularly in cases of uncertain diagnosis with MRI, which characterises most ONSM cases. PET SUV_max_ thresholds to distinguish meningioma from nonspecific uptake in other lesions need to be adapted to ONSM. ^68^Ga-DOTATOC PET improves the intraorbital lesion diagnostic approach and therefore impacts therapeutic management.

## 1. Introduction

Optic nerve sheath meningiomas (ONSMs) are benign tumours that arise from meninges around the optic nerve. The optic nerve sheath is a rare meningioma location, accounting for 1–2% of meningiomas [1], which are a frequent cause of primary optic nerve tumours [2] with variable clinical presentations and prognoses.

ONSMs most frequently occur in adults between 30 and 50 years of age and most often in females, with a sex ratio of 3:1 [2]. However, ONSMs can occur in any age group, even children. Rarely, they can be bilateral.

ONSMs’ clinical presentation can vary from no symptoms to ocular motility disturbance, proptosis, exophthalmos, and progressive vision loss via compressive optic neuropathy, as specified in ophthalmologic examinations. Papilledema might also be observed. Prognosis varies and is essentially functional, depending on location, the main risk being definitive vision loss if left untreated.

Clinical management depends on functional impairment. Some ONSMs remain asymptomatic and only need observation, whereas others need appropriate therapies. Progestin intake should always be assessed as it increases the risk of meningioma, especially if taken over a long period of time or in high doses, and should be discontinued when possible. Therapeutic management includes surgery, which comprises delicate, stereotactic radiotherapy and intensity-modulated radiation therapy (IMRT) with a risk of radiation-induced retinopathy or neuropathy. Currently, radiation therapy (RT) is the preferred treatment in cases of visual impairment.

Diagnosis relies mainly on clinical and imaging features since biopsy in this location can be deleterious and is avoided as much as possible. Typical MRI findings are the following: optic nerve enlargement with thickening of the meninges surrounding the optic nerve that can be tubular, globular, fusiform, or focal, and isointensity on T1- and T2-weighted sequences [3]. After gadolinium injection, meningiomas usually demonstrate intense enhancement. In axial MR images, the typical “tram track” sign can be observed with homogeneous enhancement of the lesion surrounding the optic nerve and no inner enhancement. In coronal MR images, the typical “doughnut sign” or the “nonenhancing dot sign” is observed [4].

However, MRI findings can be nonspecific and sometimes fail to differentiate meningiomas from their other main differential diagnoses, which include tumours such as gliomas, lymphomas, and metastases; granulomatosis such as neurosarcoidosis or tuberculosis; and optic perineuritis. In MRI, optic nerve glioma typically presents as uniform enhancement of the optic nerve, but in some cases, the nerve sheath is thickened, mimicking ONSM. In ONSM, the meningioma can sometimes invade the optic nerve and not be confined to the sheath. Furthermore, the typical tram track sign visible in ONSM may be observed in many other diseases that represent the main differential diagnoses of ONSM, such as perioptic neuritis, orbital sarcoidosis, lymphoma, and metastases [4].

Noninvasive PET imaging with ^68^Ga-DOTA-conjugated somatostatin analogues can differentiate meningiomas from their other main differential diagnoses [5] with high diagnostic accuracy.

Indeed, meningiomas are known to intensely express somatostatin receptor subtype 2A (SSTR2A), which can be targeted with radiolabelled somatostatin analogues (DOTATOC, DOTATATE, or DOTANOC), even when they are aggressive. There has been rising interest in the use of positron emitters for the diagnosis of meningiomas, whereby a high tumour-to-background ratio is sometimes able to diagnose millimetric remnant lesions [6]. Meningiomas show high ^68^Ga-DOTATOC uptake, with maximum standardised uptake values (SUV_max_) between 4.3 and 68.7 for all types of meningiomas [7]. In a recent study, the SUV_max_ was 14.3 ± 15.4 for optic pathway meningiomas, with a sensitivity and specificity of 100% for detecting ONSM [8].

It is important to note that, unlike MRI, PET is a molecular imaging technique that identifies the molecular target SSTR2 in the case of DOTATOC PET. High uptake by a lesion of DOTATOC PET reflects that the lesion has high SSTR2 expression, as revealed through receptor binding. DOTATOC PET uptake in meningiomas is well correlated with SSTR2 expression in the lesions in anatomopathological studies [9].

Only a few cases of ^68^Ga-DOTATOC PET in the diagnosis of ONSM have been reported [8,10,11,12]. To our knowledge, no case of ^68^Ga-DOTATOC PET in the follow-up for ONSM has been reported.

We report a series of 12 cases of suspected optic nerve sheath meningiomas and analyse the impact of ^68^Ga-DOTATOC PET on the diagnostic strategy for and clinical management of ONSM.

## 2. Materials and Methods

All patients who underwent ^68^Ga-DOTATOC PET in the Department of Nuclear Medicine, Marseille, APHM, between January 2020 and April 2022 for suspected ONSM were retrospectively included. ^68^Ga-DOTATOC PET was performed using an integrated PET/CT camera (General Electric Healthcare Discovery PET/CT 710) after the intravenous injection of a 2.5 MBq/kg radiotracer. A protocol of 7 min of acquisition, 1 h postinjection, was used. Images were reconstructed on a 192 × 192 matrix using the ordered subsets expectation maximisation algorithm and were corrected for attenuation using a CT transmission scan.

Standardised clinical and radiological data were recorded. When MR images were available, PET images were fused with the MR images using alignment algorithms. Hence, images could be represented as PET/MRI fused images, even if acquired with a PET/CT camera, and secondarily fused with MRI. PET examination results were classified as positive if DOTATOC uptake was considered elevated and negative if DOTATOC uptake was considered low or null by two independent nuclear physicians. The maximum standardised uptake values of the optic lesion and pituitary gland were recorded. Initially, PET was classified as positive or negative based on visual interpretation. Subsequently, an optimal threshold, based on SUV_max_, to classify PET as positive or negative was chosen via discussion amongst the authors.

No biopsies were performed. All cases were discussed in multidisciplinary meetings with a neuro-oncologist, radiotherapist, neurosurgeon, and nuclear physician. Patients were followed up by physicians, and further examinations were performed when necessary (for example, spinal tap examinations and FDG PET in cases of suspected lymphoma). Follow-up visits and multidisciplinary meetings were employed to (i) validate the diagnosis of ONSM, (ii) give the case an uncertain diagnosis, or (iii) choose other diagnoses.

Preradiation therapy (RT) volume delineation was performed on all cases by the same radiation therapist. The biological target volume (BTV) was defined using ^68^Ga-DOTATOC PET. The gross tumour target volume (GTV) was defined using MRI with at least T1-weighted gadolinium and T2 FLAIR sequences. The clinical target volume (CTV) was defined as the GTV expanded by a 3 to 5 mm margin to consider the microscopic extension of the disease. CTV could be manually modified by the radiation oncologist according to the MRI interpretation. BTV and CTV were compared in patients who were treated in our department.

This study was approved by the institutional review board of the French College of Neurosurgery (approval code: IRB00011687; date of approval: 13 May 2022). Retrospective observations required nothing other than informed consent according to French and European regulations.

### Statistical Analysis

Mean values as well as ratios were calculated using Microsoft ^®^ Excel 2023.

## 3. Results

Twelve ^68^Ga-DOTATOC PET scans for suspected ONSM were analysed. All patients presented with a unilateral optic nerve lesion in MR images. Most MRI examinations showed an isointense T1-weighted lesion with peripheral enhancement. Six examinations were considered positive due to intense ^68^Ga-DOTATOC uptake, and six exams were considered negative. Table 1 summarises the main clinical data on the reported cases with their ^68^Ga-DOTATOC results.

### 3.1. Positive ^68^Ga-DOTATOC PET

**Figure 1:**
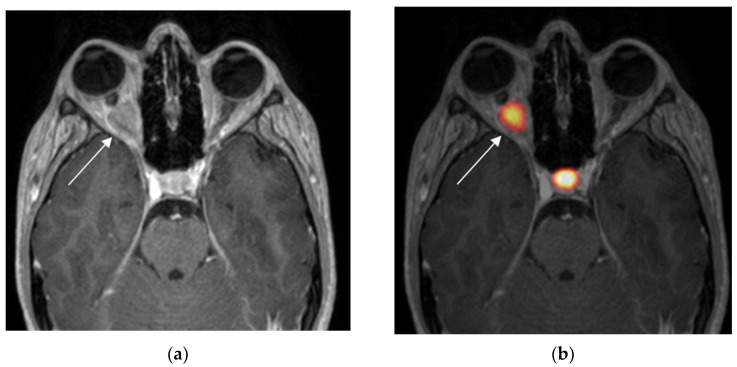
Case 1. Axial MRI T1WI (**a**) showing outer enhancement of a right intraorbital tumour and axial fused MRI, and ^68^Ga-DOTATOC PET images (**b**) showing intense tracer uptake in the right intraorbital tumour (white arrow) and intense physiologic uptake in the pituitary: tumour SUV_max_ = 11.9; pituitary SUV_max_ = 17.

**Figure 2:**
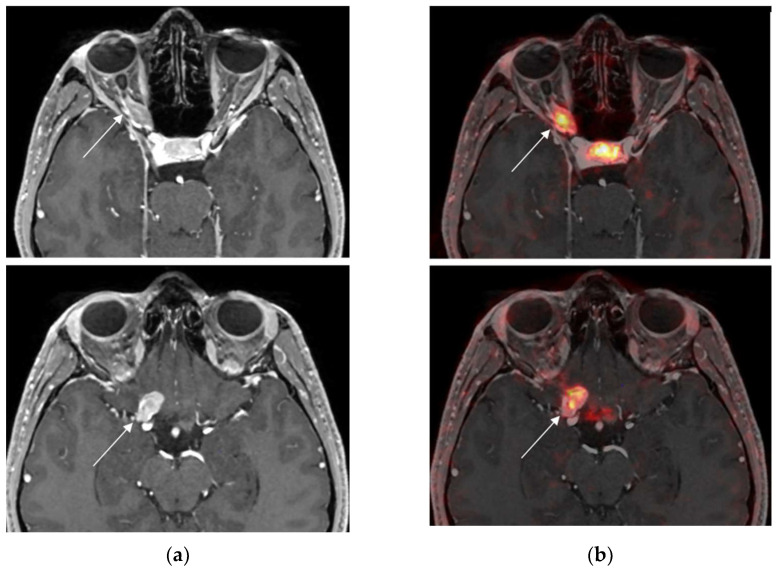
Case 2. Axial MRI T1WI (**a**) showing enlargement with enhancement of the right intraorbital (top row) and prechiasmatic tumours (bottom row) (white arrow). Axial fused MRI and ^68^Ga-DOTATOC PET images (**b**) showing intense tracer uptake in the right intraorbital tumour (white arrow): tumour SUV_max_ = 6.9; pituitary SUV_max_ = 9.4.

**Figure 3:**
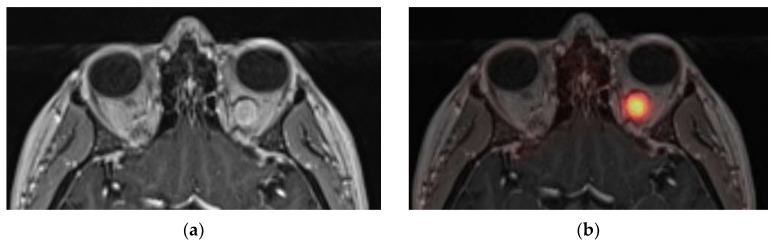
Case 4. (**a**) Axial MRI T1WI (**a**) showing outer enhancement of the left intraorbital tumour and axial fused MRI. (**b**) ^68^Ga-DOTATOC PET images showing intense tracer uptake in the left intraorbital tumour: tumour SUV_max_ = 19.6; pituitary SUV_max_ = 17.0.

Cases 1 to 6 were considered positive ^68^Ga-DOTATOC examinations by virtue of their intense radiotracer binding in the optic lesion, which is strongly suggestive of ONSM (Figure 1, Figure 2 and Figure 3).

Of these, two patients presented with progressive vision loss at onset, and two were older patients (74 and 82 years old) with rapid vision loss. One patient (case 6) had already been treated with IMRT 6 years ago for a suspected ONSM and presented 1 year ago with visual impairment of the treated eye. ^68^Ga-DOTATOC PET was performed to confirm the diagnosis of meningioma before they underwent novel radiotherapy.

One positive case had no vision loss (case 4; Figure 3). This 41-year-old woman presented with isolated retro-ocular pain. She had been under progestin hormonal treatment (chlormadinone acetate) for more than 10 years for endometriosis. Given the long-term hormone intake and the lack of visual trouble, hormonal treatment withdrawal with close ophthalmologic follow-up was proposed.

Concerning the six positive ^68^Ga-DOTATOC PET scans, the SUV_max_ values of the retained ONSMs ranged from 6.3 to 19.6, with a mean SUV_max_ = 10.8.

The SUV_max_ ONSM/pituitary ratios ranged from 0.4 to 1.1, with a mean ratio of 0.8.

Case 5 was a 74-year-old woman first referred for right blurred vision. An ophthalmologic examination revealed disc pallor and diminished vision in the right eye. MRI showed a hyperintense T2 enlargement of the intraorbital and intracanalar right optic nerves, with peripheral enhancement shown in T1-weighted images. Neuritis, lymphoma, and ONSM were suspected. The results of the medullary MRI, spinal tap, and two ^18^F-FDG whole-body and brain PET scans were negative. High-dose corticosteroid therapy had no clinical effect.

^68^Ga-DOTATOC PET showed moderate–high DOTATOC uptake with an SUV_max_ of 6.3, but the lesion/pituitary ratio was low (0.4). The absence of any FDG uptake by the lesion combined with radioclinical stable evolution and moderate ^68^Ga-DOTATOC uptake led to the diagnosis of ONSM. RT was discussed but not started for personal reasons.

### 3.2. Negative ^68^Ga-DOTATOC PET

**Figure 4:**
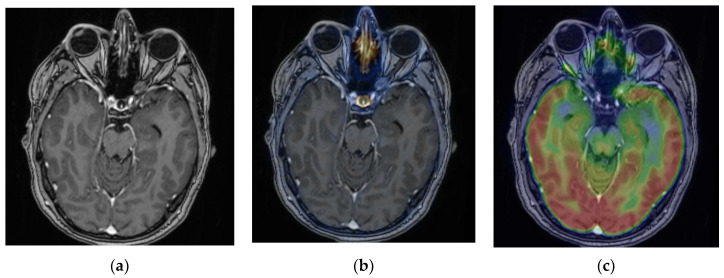
Case 7. Axial slices of T1W1 gadolinium (**a**), ^68^Ga-DOTATOC fused PET-MRI (**b**), and ^18^F-FDG fused PET-MRI (**c**) showing low DOTATOC (SUV_max_ = 3.6 for the lesion, and SUV_max_ = 10.2 for the pituitary) and moderate FDG uptake in the left optic nerve cisternal segment enhanced lesion.

**Figure 5:**
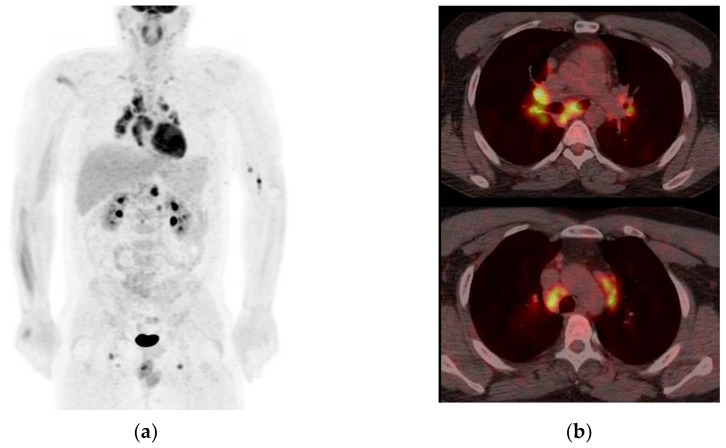
^18^F-FDG maximal intensity projection (**a**) and fused PET-CT axial slices showing multiple intense hypermetabolic mediastinal (**b**) and infra-mediastinal lymphadenopathies.

Six PET examinations were considered negative due to low ^68^Ga-DOTATOC uptake (cases 7 to 12). Four diagnoses remained unknown. In the absence of worsening vision loss, no biopsy was performed in these cases. One patient was diagnosed with sarcoidosis (case 7; Figure 4 and Figure 5). This 35-year-old man was referred for blurred vision and progressive loss of vision in the left eye. MRI showed tubular thickening of the left optic nerve in its cisternal portion extending over 2.2 cm, with isointensity on T1-weighted imaging and intense homogenous enhancement. The lesion had low ^68^Ga-DOTATOC and moderate ^18^F-FDG uptakes. Whole-body ^18^F-FDG PET highlighted marked hypermetabolic mediastinal and infra-mediastinal lymphadenopathies. The conclusion was systemic and neurologic sarcoidosis, which was confirmed with a biopsy of the inguinal node. Infliximab and corticosteroid therapy were initiated.

One patient was diagnosed with a probable cavernous malformation (case 12). This 67-year-old man was referred for brutal left retro-ocular pain and diplopia to the right lateral gaze one month before the examination. MRI showed a round heterogeneous lesion of the left orbit, with mixed signal intensity on T1- and T2-weighted images and no enhancement. ^68^Ga-DOTATOC PET was performed to rule out the diagnosis of ONSM and showed low DOTATOC uptake in the lesion, which is consistent with the diagnosis of a cavernous malformation. A repeat MRI examination three months later revealed a decrease in lesion size, suggesting previous intratumoural haemorrhage. In the absence of visual trouble, no surgical treatment was proposed.

### 3.3. Added Value of ^68^Ga-DOTATOC PET before RT in Two Positive Cases

**Figure 6:**
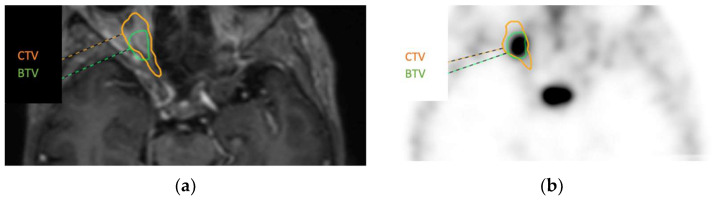
Case 3. Axial slices showing in MRI (**a**) and ^68^Ga-DOTATOC PET (**b**) the clinical target volume (CTV) and the biological target volume (BTV) of 2.5 and 1/1 cm^3^, respectively.

In two positive ONSM cases (cases 2 and 3), the BTVs obtained from ^68^Ga-DOTATOC PET and the CTVs obtained from MRI were compared. Both measures were defined by the same radiation therapist. The BTVs obtained for cases 2 and 3 were 2.5 cm^3^ and 1.1 cm^3^, respectively, whereas the CTVs were 6.8 and 2.5 cm^3^, respectively (see Figure 6 for case 3). In both cases, the lesion volume was overestimated with MRI because of the difficulty of tumour delineation in the optic nerve using this technique.

## 4. Discussion

The optic sheath nerve is a rare location for meningiomas, which explains the scarce cases with ^68^Ga-SSTR ligand PET in the literature. Meningiomas usually have high SSTR2A expression and hence high ^68^Ga-DOTATOC uptake, in contrast with most tumours or inflammatory lesions that can show low, nonspecific ^68^Ga-DOTATOC uptake [5].

### 4.1. Is Positive Radiolabelled SSTR2A-PET True Positive?

Radiolabelled SSTR2A-PET is a noninvasive tool to identify the presence of SSTR2A. A high uptake by the lesion in PET reflects that the lesion has high SSTR2 expression that has bound the tracer, which has been confirmed in anatomopathological studies [9].

Other tumours can exhibit high SSTR2A expression, such as pituitary adenoma or neuroendocrine tumours, but are not usually found in the optic pathway sheath or orbital location. Rare cases of NET metastases in the optic pathways have been described, but the primary tumour is usually noisy, and the clinical context helps to identify it.

Hence, intraorbital tumours with high ^68^Ga-DOTATOC uptake can be concluded to be meningiomas with a high predictive positive value. When we see a high uptake, we do not expect a false positive in the optic pathways. Klingenstein et al. reported 100% specificity in their study on optic pathway tumours [8]. But the challenge is to find the optimal value that would define the uptake as high and, therefore, to conclude that it is a meningioma with 100% specificity. In the literature, it seems that “high uptake” relies most often on the visual interpretation of nuclear physicians, and no value has been proposed to define high uptake in PET, especially when applied to the optic nerve. In Table 2, we summarise positive radiolabelled SSTR2A-PET cases from the present study and from the literature in which detailed data were available.

In the present case series, the SUV_max_ values of lesions with a obtained diagnosis of ONSM ranged from 6.3 to 19.6 (mean SUV_max_ = 10.8), which are close to results found in the literature (mean SUV_max_ = 14.3 in a series of optic pathway meningiomas, also including sphenoidal and tuberculum sellae meningiomas) [8] and slightly higher than those in another series (with a median SUV_max_ of 5.6) [12].

### 4.2. True Negative Radiolabelled SSTR2A-PET

Low tracer uptake can be found in many tumours or inflammatory lesions, as illustrated in Table 3. Table 3 summarises cases from our experience and from the literature in which low uptake was observed in radiolabelled SSTR2A-PET of non-meningioma optic nerve lesions (see true negative). In addition, some gliomas have been found to express SSTR2A and can have a low nonspecific ^68^Ga-DOTA uptake that is not necessarily correlated with SSTR2A expression (tumour SUV_max_ = 2.25 ± 1.33 in a study consisting of 19 gliomas) [13].

A threshold of 2.3 SUV_max_ has been suggested by some authors [9] to discriminate between meningioma tumoural and nontumoural tissue. In that study, this SUV_max_ delineated meningiomas in PET before RT but did not seem able to differentiate meningiomas from other lesions or distinguish certain types of meningiomas, such as ONSM. In the same study by Rachinger and colleagues, 9/82 lesions with SUV_max_ > 2.3 were identified as not being meningiomas with a specificity of 73.5% [9]. In our study and in the literature, true negatives in PET have SUV_max_ values ranging from 1.7 to 3.6 (see Table 3). Hence, in the presence of low radiotracer uptake in PET, an optic nerve lesion should not be concluded to be a meningioma.

### 4.3. False Negative ^68^Ga-DOTATOC PET

Rachinger et al. [9] observed that 8/81 (approximately 10%) of meningiomas had an SUV_max_ of <2.3. In a series of 8 patients with ten suspected ONSMs, Graef et al. reported one histologically confirmed PET-negative ONSM with an SUV_max_ of 1.7 [12]. However, in their series, they considered PET-positive optic lesions to be those with SUV_max_ values ranging from 2.6 to 7.8, while we would have considered a lesion with an SUV_max_ of 2.6 in PET to be negative. In Graef et al.’s study [12], in a series of 10 suspected ONSM cases, only 3 cases were histologically proven, including the case with an SUV_max_ of 1.7. It was not detailed which other cases benefited from a biopsy. Klingenstein et al. [8] reported two cases of probable ONSM considered negative in PET but with no histological confirmation. A positive diagnosis of ONSM was made from radio-clinical typical findings (see Table 3).

Therefore, if a lesion has low ^68^Ga-DOTATOC uptake, the probability of a negative ^68^Ga-DOTATOC meningioma is low but cannot be completely excluded. A small percentage of meningiomas do not, or weakly, express SSTR2A. Special attention should be paid to small lesions. They may cause false negatives because of the partial volume effect due to the limited spatial resolution of PET. The spatial resolution depends on several parameters, such as the background-to-lesion ratio, PET camera characteristics, and PET acquisition parameters. Graef et al. [12] reported a 4 mm ONSM with a nearly absent PET ^68^Ga-DOTATOC signal.

Additionally, rare, isolated cases of negative immunohistochemical SSTR2A meningioma have been reported [14], with subsequently low-level avidity in ^68^Ga-SSTR2A radiolabelled PET.

Table 4 summarises the literature results on SSTR2A expression in meningioma using polymerase chain reaction (PCR) and immunohistochemical staining (IHC). SSTR2A negative expression was close to 0% in most studies using PCR and varied from 8 to 73% using IHC. SSTR2A expression was demonstrated to be stronger in the skull base and meningothelial meningiomas, which correspond to the most frequent subtype and location of ONSM [15,16].

### 4.4. What Is the Optimal Threshold to Differentiate ONSM from Non-Meningioma Optic Nerve Lesions?

**Figure 7:**
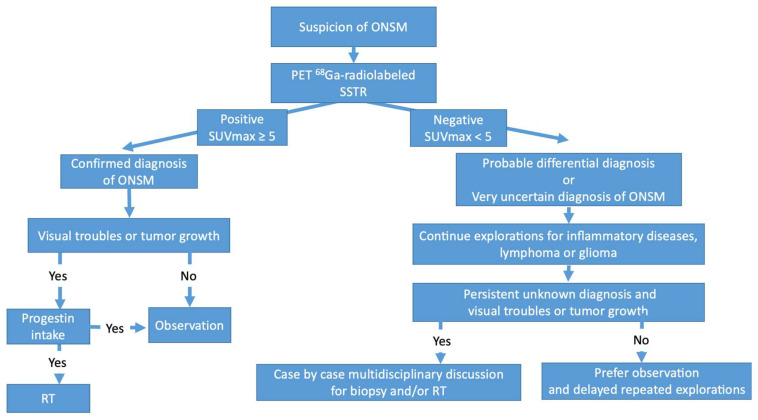
Authors’ proposed organisational chart for the diagnosis and management of optic nerve sheath meningioma (ONSM) using ^68^Ga-DOTATOC positron emission tomography (PET). SUV_max_ = maximum standardised uptake value, RT = radiotherapy, and SSTR = somatostatin receptor.

Based on the present study and earlier studies, an SUV_max_ of ≥5 could be an appropriate and specific threshold to confirm the diagnosis of ONSM with high specificity and avoid biopsy (Figure 7). The use of the SUV_max_ lesion/pituitary ratio may be helpful to improve the ^68^Ga-DOTATOC power of discrimination between meningiomas and other lesions. Nevertheless, the role of the lesion/pituitary ratio still needs to be specified.

### 4.5. What Is the Place for Radiolabelled SSTR2A-PET in ONSM?

In ONSM, as with a complex skull base location, given the surgical biopsy risk and morbidity, histological proof of meningioma is not systematically required to treat a patient with radiotherapy or stereotactic radiosurgery. Typical MRI could be considered sufficient for choosing the treatment but probably leads to the misdiagnosis and mistreatment of some cases, increasing the risk of treatment failure. In ONSM, ^68^Ga-DOTATOC PET positivity could reinforce the diagnosis of meningioma before RT.

Furthermore, ^68^Ga-DOTATOC PET might help define the target volume for RT when tumour delineation is difficult using solely MRI or CT, as already suggested in the literature [9,12]. Therefore, ^68^Ga-DOTATOC PET could impact the management of intraorbital tumours by reinforcing the radiotherapy indications in cases of positivity or avoiding radiotherapy in cases of diagnostic uncertainty. The authors of this paper suggest using ^68^Ga-DOTATOC PET before treating with radiotherapy or stereotactic radiosurgery for nonhistologically proven meningioma, particularly in cases of uncertain diagnosis with MRI, as is true for most ONSMs (Figure 7). In the case of long-term progestin intake, hormonal substitution discontinuation with close monitoring should be preferred over RT [28].

Negative ^68^Ga-DOTATOC PET leads to the suspicion of a rare meningioma with negative or low SSTR2A expression and a differential diagnostic process. RT should, at first, be avoided. Complementary explorations for lymphoma, inflammatory diseases, and glioma should be preferred. Therefore, in cases of uncertain diagnosis with visual stabilisation and lack of tumour progression, observation with iterative explorations should be started. In contrast, in cases of visual impairment or tumour progression, biopsy should be considered: the decision between lesion biopsy and RT should be discussed in a multidisciplinary manner.

Volume delineation is a central issue in radiation oncology. It can sometimes be challenging when tumour delineation is not obvious in morphological examinations such as MRI and CT. ^68^Ga-DOTATOC PET has been proposed as a useful tool to help radiation therapists define a target volume using PET, named BTV [9,12]. Tumours that have a high lesion-to-background ratio in PET, such as meningiomas, are particularly good candidates for the use of BTV. As opposed to BTV, the gross tumour target volume (GTV) and the clinical target volume (CTV) are defined using MRI or CT. An uncertain GTV delineation requires manually adapting the CTV to ensure that all the tumoural tissue is englobed within the targeted volume, including the doubtful tissue observed in MRI. Defining the BTV using ^68^Ga-DOTATOC PET is a much less uncertain measure and includes the whole disease extent without a margin, as with the CTV.

In the present study, ^68^Ga-DOTATOC PET was helpful in improving tumour volume delineation before RT, leading to a decreased applied radiation dose, which is in accordance with previous studies [12,29]. In a series of 10 ONSM patients, Graef et al. [12] found that PET/MRI enabled the avoidance of uncertainty and the inclusion of additional safety margins for radiation planning in 7/10 lesions. Stade et al. [29] concluded that ^68^Ga-DOTATOC PET helped spare normal tissue from radiation therapy in 10 skull base meningiomas, especially by reducing the dose to organs at risk.

### 4.6. Limitations

Even though this study is, to our knowledge, the largest study on ^68^Ga-DOTATOC PET for suspected ONSM, only 12 cases were reported in this monocentric study. It was limited by the rarity of ONSM (comprising 1% of all meningiomas) and by the lack of histological confirmation, as in most ONSM studies.

Another limitation is that apart from biopsy, there is a lack of a clear gold-standard method for ONSM diagnosis.

In the present study, high DOTATOC uptake led to a positive diagnosis of ONSM. This could be interpreted as a limitation and could indicate the presence of false positives. However, diagnoses were made in concordance with clinical and MRI data after multidisciplinary discussion and with a compatible follow-up. DOTATOC PET is a molecular technique that detects SSTR2A. Since other tumours that highly express SSTR2A are not found in the optic nerve, if the optic lesion presented a high SSTR2A level in PET, the PET was considered positive.

Concerning negative examinations, two diagnoses of tumours other than meningiomas were made (sarcoidosis and cavernous malformation), while four diagnoses remained unknown. These four diagnoses could have been false negatives (ONSMs with low SSTR2).

Larger series and new tools for meningioma diagnosis are required to specify the desirability of ^68^Ga-DOTATOC PET for this indication, particularly for ^68^Ga-DOTATOC PET-negative lesions.

## 5. Conclusions

As illustrated in this study, ^68^Ga-DOTATOC PET is a noninvasive, powerful, and still underused tool in the diagnostic workup of intraorbital tumours, especially ONSMs.

ONSMs are frequent primary optic nerve tumours, but their diagnosis is challenging due to nonspecific clinical or MRI findings and a lack of histology on this specific location. Meningiomas usually have high SSTR2A expression and, hence, high ^68^Ga-DOTATOC uptake in contrast with other tumours or inflammatory lesions that can show low, nonspecific ^68^Ga-DOTATOC uptake. Previously reported SUV_max_ cut-offs to differentiate meningioma from non-meningioma are probably not adapted to ONSM and do not seem specific enough. Based on the present series, an SUV_max_ threshold of 5 seems most relevant for the diagnosis of ONSM.

The role of the lesion/pituitary SUV_max_ ratio should be specified. In this case series, ^68^Ga-DOTATOC PET improved the intraorbital lesion diagnostic approach and therefore impacted therapeutic management, identifying 6 ONSMs out of 12 intraorbital lesions. Negative ^68^Ga-DOTATOC PET should lead to an uncertain diagnosis of meningioma and the possibility of differential diagnosis requiring complementary investigations and possible biopsy. Moreover, ^68^Ga-DOTATOC PET may be helpful in tumour volume delineation during radiation therapy planning.

## Figures and Tables

**Table 1:** Summary of the reported cases with ^68^Ga-DOTATOC PET results and impacts on further management or treatment. F = female; M = male.

Case	Age (Years), Sex	Main Symptoms	^68^Ga- DOTATOC PET	SUV_max_	Lesion/Pituitary Ratio	Obtained Diagnosis	Treatment
1	17, F	Progressive vision loss and exophthalmos	Positive	11.9	0.7	ONSM	Radiotherapy
2	38, F	Progressive loss of vision	Positive	6.9	0.8	ONSM	Radiotherapy
3	82, F	Rapid vision loss	Positive	11.0	0.9	ONSM	Radiotherapy
4	41, F	Retro ocular pain and no vision loss	Positive	19.6	1.1	ONSM	Observation and hormonal treatment withdrawal
5	74, F	Blurred vision and rapid vision loss	Positive	6.3	0.4	ONSM	Radiotherapy proposed
6	59, F	Recent vision loss after tomotherapy 6 years ago	Positive	9.1	0.8	ONSM	Radiotherapy
7	35, M	Blurred vision and progressive vision loss	Negative	3.6	0.3	Sarcoidosis	Corticosteroid therapy and then infliximab
8	22, F	Progressive vision loss and history of cranial nerve palsy (III and VI)	Negative	2.2	0.2	Unknown	Follow-up
9	38, F	Visual discomfort and narrowing of the visual field without vision loss	Negative	2.1	0.3	Unknown	Follow-up
10	25, M	No vision loss and fortuitous discovery of papilledema	Negative	3.3	0.1	Compressive neuropathy of unknown origin	Follow-up
11	57, F	Progression vision loss	Negative	1.2	0.1	Uncertain, possible glioma	Follow-up
12	67, M	Brutal retro-ocular pain and diplopia	Negative	1.5	0.1	Cavernous malformation	Follow-up

**Table 2:** Cases considered positive in radiolabelled SSTR2A-PET from our series and from the literature with corresponding main symptoms. Standardised maximal uptake (SUV_max_) values are given. NA = not available. F = female; M = male. The term “optic nerve meningioma” was chosen instead of ONSM in the two cases from the study by Klingenstein et al. [8] in which the meningiomas were extended outside of the orbit (possible secondary ONSM).

Study	Age (Years), Sex	Main Symptoms	Radiolabelled SSTR2A-PET	SUV_max_	Obtained Diagnosis	Treatment
Current Study Horowitz et al.	17, F	Progressive vision loss and exophthalmos	Positive	11.9	ONSM	Radiotherapy
	38, F	Progressive loss of vision	Positive	6.9	ONSM	Radiotherapy
	82, F	Rapid vision loss	Positive	11.0	ONSM	Radiotherapy
	41, F	Retro-ocular pain and no vision loss	Positive	19.6	ONSM	Observation and hormonal treatment withdrawal
	74, F	Blurred vision and rapid vision loss	Positive	6.3	ONSM	Radiotherapy proposed
	59, F	Vision loss after tomotherapy 6 years ago	Positive	9.1	ONSM	Radiotherapy
Dolar Bilge et al., 2020 [11]	16, M	Three-year history of proptosis	Positive	Visually high but NA	ONSM	Radiotherapy
Al Feghali et al., 2018 [10]	28, F	Progressive temporal vision loss over a year	Positive	10.8	ONSM	Radiotherapy
Klingenstein et al. [8]	29, F	Photopsia	Positive	6	ONSM	Radiotherapy
	50, F	Lower vision	Positive	12.6	Optic nerve meningioma	Radiotherapy and surgery
	57, M	Lower vision	Positive	17.6	Optic nerve meningioma	Cyberknife

**Table 3:** Cases considered negative in radiolabelled SSTR2A-PET from our series and from the literature with corresponding main symptoms and standardised maximal uptake (SUV_max_) values. NA = not available. F = female; M = male.

Study	Age (Years), Sex	Main Symptoms	Radiolabelled SSTR2A-PET	SUV_max_	Obtained Diagnosis	Treatment
Current study by Horowitz et al.	35, M	Blurred vision and a progressive vision loss	True negative	3.6	Sarcoidosis	Corticosteroid therapy and then infliximab
	67, M	Brutal retro-ocular pain and diplopia	True negative	1.5	Cavernous malformation	Follow-up
Klingenstein et al., 2015 [8]	61, M	Lower vision	True negative	3.2	Gastric carcinoma metastasis	Cyberknife and chemotherapy
	66, F	Lower vision	True negative	1.7	Leukaemic infiltration	Chemotherapy
	48, F	Double vision	True negative	1.4	Histologically proved inflammatory collagenous connective tissue	Corticosteroid therapy
Graef et al., 2021 [12]	NA	NA	False negative	1.7	Histologically confirmed ONSM (4 mm lesion)	Radiotherapy
Klingenstein et al., 2015 [8]	33, F	Lower vision	Non-histologically confirmed false negative	4.1	ONSM	Radiotherapy
	44, F	Lower vision and headache	Non histologically confirmed false negative	3	ONSM	Radiotherapy
Current study by Horowitz et al.	22, F	Progressive vision loss and history of cranial nerve palsy (III and VI)	Unknown	2.2	Unknown	Follow-up
	38, F	Visual discomfort and narrowing of the visual field without vision loss	Unknown	2.1	Unknown	Follow-up
	25, M	No vision loss and fortuitous discovery of papilledema	Unknown	3.3	Compressive neuropathy of unknown origin	Follow-up
	57, F	Progression vision loss	Unknown	1.2	Uncertain, possible glioma	Follow-up

**Table 4:** Somatostatin receptor type 2A (SSTR2A) expression in meningiomas by method used (IHC or PCR). IHC = immunohistochemistry; PCR = polymerase chain reaction. NA = not available.

Study		Behling et al., 2022 [17]	Dijkstra et al., 2018 [18]	Boulagnon-Rombi et al., 2017 [19]	Graillon et al., 2017 [15]	Menke et al., 2015 [20]	Silva et al., 2015 [21]	Agaimy et al., 2014 [22]	Barresi et al., 2008 [23]	Durand et al., 2008 [24]		Arena et al., 2004 [25]	Schulz et al., 2000 [26]	Dutour et al., 1998 [27]
Number of tumours		726	148	127	50	176	60	68	35	22	26	42	40	20
Method		IHC	IHC	IHC	PCR	IHC	IHC	IHC	IHC	IHC	PCR	PCR	IHC	PCR
SSTR2A expression	Negative	0.6%	0%	5%	0%	0%	0%	13%	26%	32%	0%	21%	28%	0%
	Low	8%	18.2%	NA	<25%	NA	73%	13%	23%	41%	NA	NA	23%	NA
	Used scale for low expression definition	1–4 *	Weak/focal staining 1/3	−	+/+++ **	−	+/+++	+/+++	1–4 *	+/− vs. +	−	−	+/+++	−

* Barresi et al. [23]. Scale: 1–4. ** > 0.01 SSTR2A copy/BetaGus copy.

## Data Availability

The authors have full access to the data, and the data may be available on request.
